# Immunogenetic response of the bananaquit in the face of malarial parasites

**DOI:** 10.1186/s12862-019-1435-y

**Published:** 2019-05-22

**Authors:** Jennifer Antonides, Samarth Mathur, Mekala Sundaram, Robert Ricklefs, J. Andrew DeWoody

**Affiliations:** 1 Department of Forestry and Natural Resources, Purdue University, 715 W. State Street, Pfendler Hall 141, West Lafayette, IN 47907 USA; 2 Department of Biological Sciences, Purdue University, 915 W. State St, Indiana, USA; 3 Department of Biology, University of Missouri, 1 University Blvd, St. Louis, MO USA

**Keywords:** Major histocompatibility complex, Toll-like receptor, Haemosporidian parasites, Avian malaria

## Abstract

**Background:**

In the arms race between hosts and parasites, genes involved in the immune response are targets for natural selection. Toll-Like Receptor (TLR) genes play a role in parasite detection as part of the innate immune system whereas Major Histocompatibility Complex (MHC) genes encode proteins that display antigens as part of the vertebrate adaptive immune system. Thus, both gene families are under selection pressure from pathogens. The bananaquit (*Coereba flaveola*) is a passerine bird that is a common host of avian malarial parasites (*Plasmodium* sp. and *Haemoproteus* sp.). We assessed molecular variation of TLR and MHC genes in a wild population of bananaquits and identified allelic associations with resistance/susceptibility to parasitic infection to address hypotheses of avian immune response to haemosporidian parasites.

**Results:**

We found that allele frequencies are associated with infection status at the immune loci studied. A consistent general trend showed the infected groups possessed more alleles at lower frequencies, and exhibited unique alleles, compared to the uninfected group.

**Conclusions:**

Our results support the theory of natural selection favoring particular alleles for resistance while maintaining overall genetic diversity in the population, a mechanism which has been demonstrated in some systems in MHC previously but understudied in TLRs.

**Electronic supplementary material:**

The online version of this article (10.1186/s12862-019-1435-y) contains supplementary material, which is available to authorized users.

## Background

The survival of any multicellular organism is dependent upon an effective immune response to ward off invaders. The genes involved in this defense are thus targets for natural selection, and multifaceted mechanisms underlie vertebrate immune gene evolution driven under selective pressure from parasites [1–3].

Two major immune gene families in the vertebrate immune system are the Major Histocompatibility Complex (MHC) of the adaptive immune system and the Toll-like Receptor (TLR) family of the innate immune system. The MHC is a genetically diverse multigene family that plays a vital role: the host’s MHC receptor molecules bind peptides (antigens) produced by pathogens. MHC molecules then display the antigen on the cell’s surface for recognition by T-cells and subsequent attack on the foreign invader [4]. Therefore, an individual’s MHC genotype governs its ability to detect particular pathogens, affecting its susceptibility to parasitic infection and specific diseases [5, 6].

The MHC has long been the subject for the study of maintenance of genetic diversity by balancing selection. Balancing selection may be driven by negative frequency-dependent selection, in which rare alleles confer a selective advantage, and/or heterozygote advantage, in which heterozygotes are more fit than homozygotes [7, 8]. Additionally selection may fluctuate over space and time [9]. Heterozygote advantage implies that the highest number of MHC alleles would confer the highest fitness by allowing for recognition of the largest diversity of pathogens. However, too much MHC variability can result in removal of T-cells capable of distinguishing “self” from “non-self” and increase the chance of autoimmune disease, potentially conferring higher fitness to individuals with intermediate MHC diversity (the “optimality” hypothesis) [10, 11].

While studies of the impacts of immunogenetic variation on a host’s response to pathogens have mainly focused on MHC genes, genetic variability in other immune loci such as TLRs may also play an important role [12]. TLRs recognize conserved pathogen-associated molecular patterns (PAMPs) derived from different classes of microbes, and upon binding a foreign ligand, induce a signal cascade for the inflammatory response [13]. Evidence suggests TLRs are not as polymorphic as MHC as they are dominated by stabilizing or purifying selection due to functional constraints, but positive selection has been shown in putative ligand-binding regions of TLRs [14–16]. TLRs are therefore under similar host-parasite selective pressures as MHC, so genetic variability at these loci likely affect a host’s resistance, and TLR diversity of avian species of conservation concern has begun to be explored [17, 18].

In an attempt to further address associations between host immune gene diversity and susceptibility to parasites, we studied a wild population of bananaquits (*Coereba flaveola*). The bananaquit, a non-migratory songbird commonly found in the Caribbean and parts of South America and Mexico, lives in a variety of habitats, including forests, shrublands, and human environments such as parks. It shares the characteristics of many “finch-like” tanagers, such as a small body size, colorful plumage, and has a primarily nectivorous diet [19]. The bananaquit is a common host of Haemosporidian parasites (*Plasmodium* spp., and *Haemoproteus* spp.), blood-borne pathogens vectored by dipteran insects. These parasites cause avian malaria, which affects a wide range of birds worldwide and can impact the host’s fecundity, lifespan, and survivorship [20–22]. One would expect the genetic makeup of MHC and TLR genes in a bananaquit population to evolve in concert in the face of these parasites. In particular, any adaptation in TLR genes would likely be evidenced in the variable extracellular leucine-rich repeat region (LRR) associated with detection of the particular PAMPs. In MHC genes, we would expect evolution of exons 2 and 3 in MHC Class I genes and exon 3 in MHC Class II genes, which encode the peptide-binding regions.

Using a population of bananaquits with different infection statuses sampled from.

Guanica Forest in Puerto Rico, we attempted to identify TLR and MHC allelic associations with resistance or susceptibility to parasitic infection. This population was shown to be subject to infection by three genetically distinct lineages of *Haemoproteus*, the host specialist LA07 and host generalists OZ02 and OZ21, and the overall parasite prevalence of the population over the time of the sampling period averaged 51% [23, 24]. We grouped these individuals by infection status and use a pooled amplicon sequencing approach to identify alleles present at MHC and TLR loci. We expected to find different allele frequencies and sequence divergence among the groups, and looked for supporting evidence of mechanisms of selection. If at a particular locus, uninfected bananaquits exhibit higher allelic diversity compared to infected bananaquits, the mechanism of heterozygote advantage would be supported for that locus. Alternatively, if uninfected bananaquits exhibit lower allelic diversity, frequency-dependent selection or positive selection for particular genes that confer resistance is more likely explanation.

## Results

### Sample collection & processing

A total of 99 bananaquits were collected from Guanica Forest and used in this study. Of those, 45 were uninfected, 41 were infected with the LA07 strain of *Haemoproteus* sp., and 13 were infected with various strains of *Haemoproteus* sp. (either OZ02 or OZ21 lineages) (Additional file 1: Table S1).

### Pooled Sequencing & Sequence Processing

Ultimately, we successfully amplified, indexed, equimolarly pooled, sequenced, and called alleles for 5 immune loci in the same 99 bananaquit individuals:45 in the uninfected group (called UNI) 41 in the group infected with host-specialist LA07 (called LA07), and 13 in the group infected with either OZ02 or OZ21 (called INF). (UNI = uninfected, LA07 = infected with host-specialist LA07 strain, or INF = infected with OZ02 and OZ21). The 5 immune loci included two portions of MHC1-UAA gene (referred to here as MHC1-UAA-1 and MHC1-UAA-2) and 3 TLRs (TLR1A, TLR2B, and TLR7–1).

Upon sequencing, 15,845,214 raw reads were produced. After merging sequence reads and filtering by quality and length, 6,093,555 reads were retained (mean of 1,218,711 reads/locus; mean of 12,310 reads/locus/individual).

### Allele characterization

All of the five loci were polymorphic, with a range of 3–14 alleles (Table 1). In the UNI group 2–5 alleles were seen among the loci, the LA07 group showed between 1 and 7 alleles per locus, and the INF group showed 3–7 alleles.

**Table 1:** Summary of loci/amplicons sequenced

			GROUP											
			UNI				LA07				INF			
			n = 90 (45 individuals)				n = 82 (41 individuals)				*n* = 26 (13 individuals)			
		Total # Alleles	# Alleles	# Unique Alleles	Amplicon Depth (# reads)	Avg. Allele Depth	# Alleles	# Unique Alleles	Amplicon Depth (# reads)	Avg. Allele Depth	# Alleles	# Unique Alleles	Amplicon Depth (# reads)	Avg. Allele Depth
LOCUS	TLR1A	7	4	0	3310	828	5	0	1711	342	5	2	2709	542
	TLR2B	14	5	0	2467	493	7	2	2387	341	7	7	1465	209
	TLR7	7	5	0	3216	643	5	0	3741	748	7	2	3695	528
	MHC1-UAA-1	3	3	0	3816	1272	1	0	3442	3442	3	0	3616	1205
	MHC1-UAA-2	4	2	0	4382	2191	4	0	3875	969	4	0	2794	699

Loci/amplicons and their presumptive alleles by group (phred score > 20, amplicon depth > 1000, allele depth > 100, minor allele frequency = 4%). *UNI* Uninfected group, *LA07* infection with LA07 strains (host specialist), *INF* various infection strains other than LA07

Alleles within each locus were the same length and among the loci ranged from 363 to 384 nucleotides. There were between 2 and 16 polymorphic sites in each locus corresponding to a proportion of polymorphic sites of 0.005–0.042 (Table 2).

**Table 2:** Summary of sequence variation across loci/amplicons

	# Alleles	Length	# Polymorphisms	# Polymorphisms / Length	π
TLR1A	7	363	5	0.014	0.006
TLR2B	14	384	16	0.042	0.013
TLR7	7	372	8	0.022	0.008
MHC1-UAA-1	3	371	2	0.005	0.004
MHC1-UAA-2	4	364	4	0.011	0.006
Mean over loci	7	371	7	0.019	0.007
St. Dev. over loci	4	8	5	0.014	0.004
Range over loci	3–14	363–384	2–16	0.005–0.042	0.004–0.013

Presumptive alleles detected at each locus/amplicon and their overall genetic variation. π = nucleotide diversity (average pairwise differentiation between unique allele sequences)

A large portion of TLR genes are conserved but the recognition of particular PAMPs of parasites occurs by particular polymorphic amino acids of the extracellular N-terminal domain containing leucine-rich repeats (LRR). Primers were therefore designed for each TLR such that coding regions for a portion of the LRR domain were amplified. In the three TLRs successfully sequenced in this study, TLR1A, TLR2B, and TLR7–1, the levels of polymorphism observed suggest at least a portion of these pathogen recognition areas were amplified, which enhances our chances of detecting signatures of selection.

In MHC Class I genes, the peptide binding groove is formed when the α1 and α2 domains (encoded by exons 2 and 3 of the gene) of the alpha chain fold in to form a pocket where the ligand is recognized. The particular amino acids lining the walls of this groove are highly polymorphic to recognize various ligands, and are encoded by multiple short regions spaced throughout the nucleotide sequence that encodes the α1 and α2 domains. Because the amino acids which line the groove depends on the 3D structure of the protein, those precise nucleotide regions cannot be determined by examination of the genome sequence alone. In the case of the MHC Class I gene that was successfully amplified in this study (MHC1-UAA), the primers were designed such that two separate regions of the genome sequence encoding the α1 and α2 domains would be flanked in an attempt to capture at least a portion of the peptide-binding region. The first locus, MHC1-UAA-1, has a length of 371 nucleotides: the first 333 bp code for 111 amino acids, then there is a stop codon, and then 35 more nucleotides of an intronic region. The second locus, MHC1-UAA-2, has a length of 364 nucleotides: the first 1–312 nucleotides code for 104 amino acids, then there is a stop codon, and then 48 nucleotides of an intronic region. Unfortunately both regions (MHC1-UAA-1 and MHC1-UAA-2) spanned by our primers showed no or little polymorphism in either coding or non-coding (intron) regions.

### Statistical analyses

#### Frequency-based analyses

**Fig. 1:**
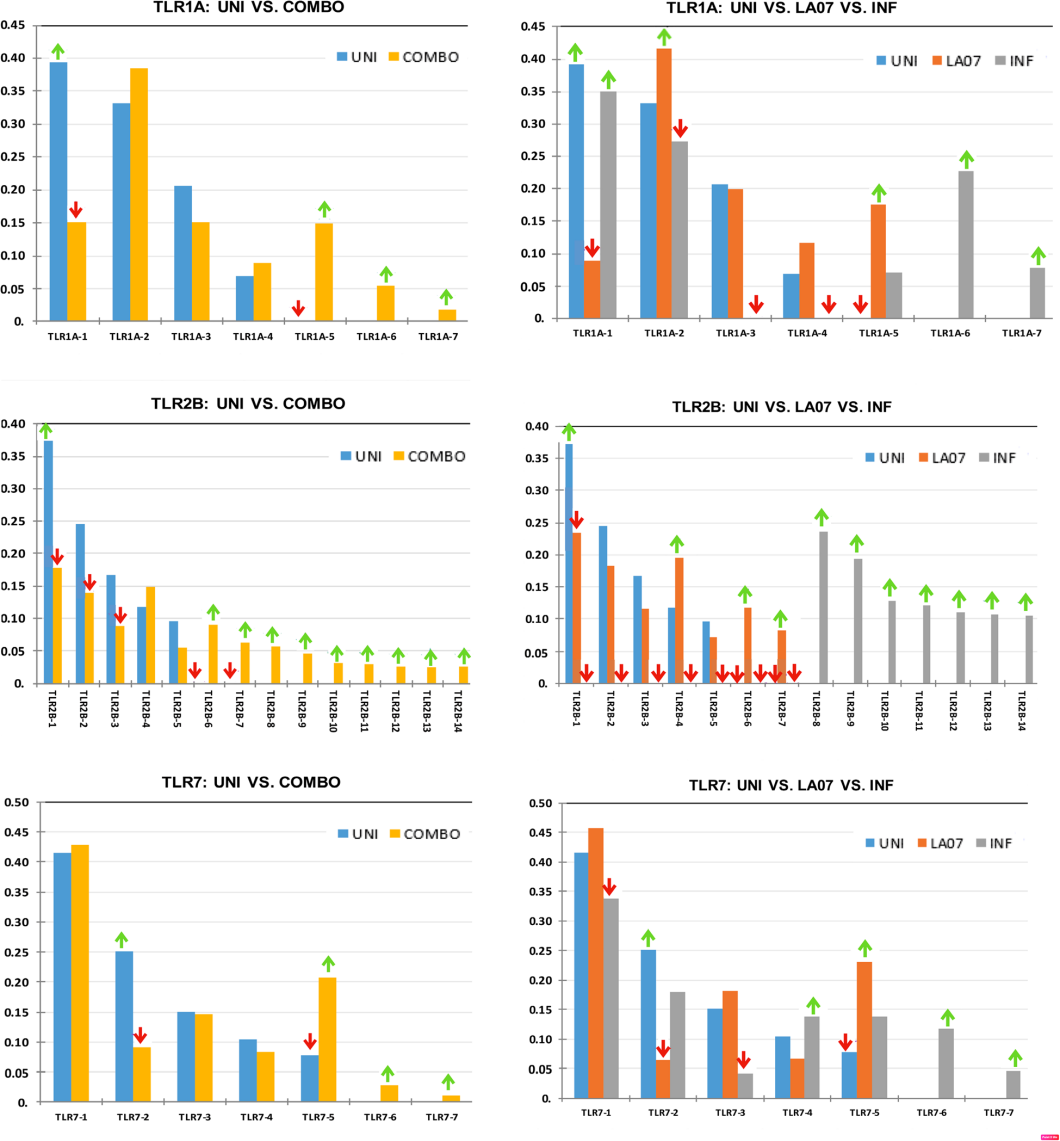
Allele frequency differences at TLR loci between experimental groups. All group/locus correlation/association tests are overall significant at among groups based on Chi-square test and/or Fisher’s exact test. Alleles with significantly higher (green up-arrow) or lower (red down-arrow) frequencies based on the 95% confidence interval of the null distribution upon resampling. *UNI* Uninfected group, *LA07* infected with LA07 strains, *INF* infected with various strains other than LA07. COMBO represents combined infected groups (the weighted average of LA07 and INF)

**Fig. 2:**
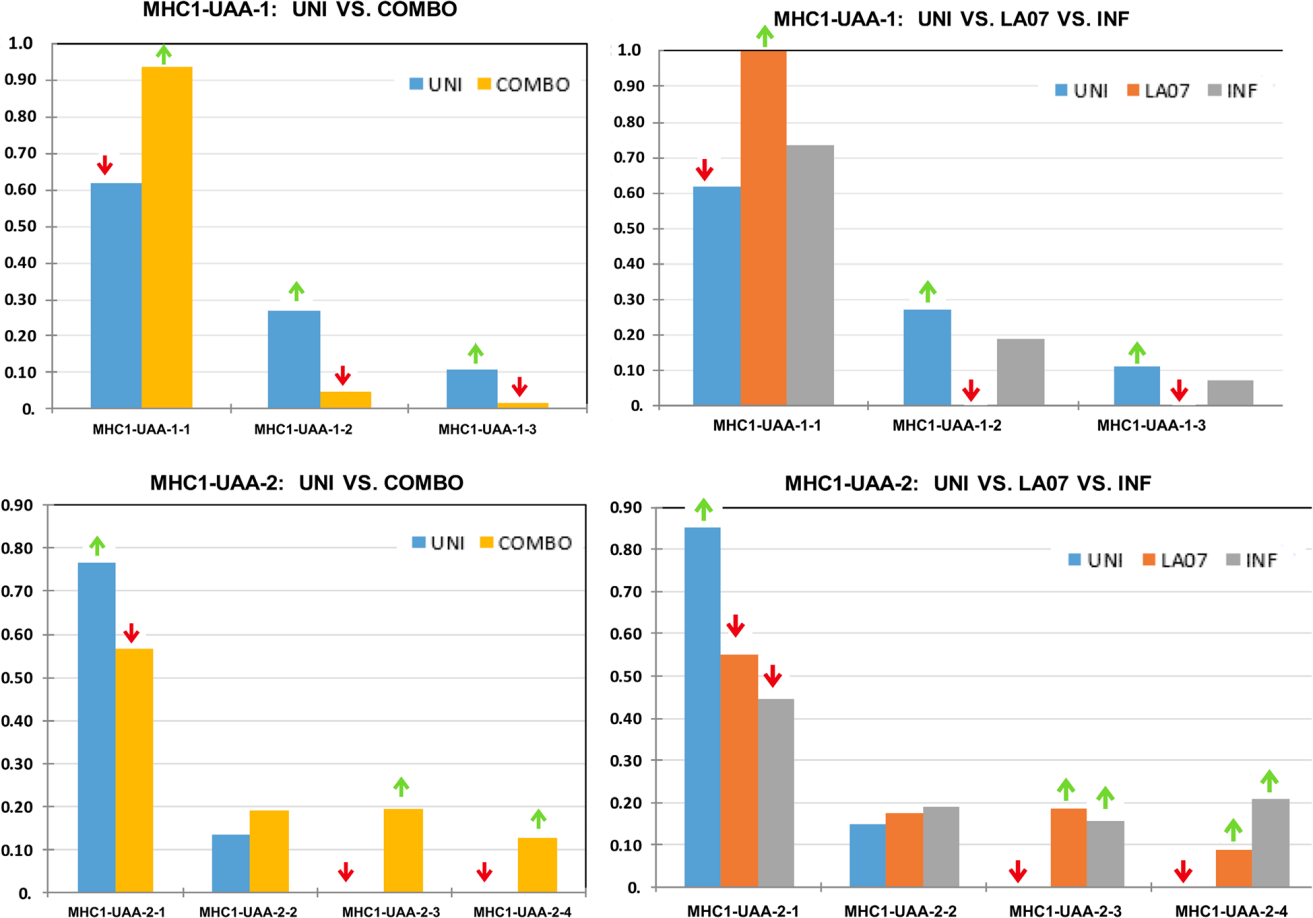
Allele frequency differences at MHC loci between experimental groups. All group/locus correlation/association tests are overall significant at among groups based on Chi-square test and/or Fisher’s exact test. Alleles with significantly higher (green up-arrow) or lower (red down-arrow) frequencies based on the 95% confidence interval of the null distribution upon resampling. *UNI* Uninfected group, *LA07* infected with LA07 strains, *INF* infected with various strains other than LA07. COMBO represents combined infected groups (the weighted average of LA07 and INF)

**Fig. 3:**
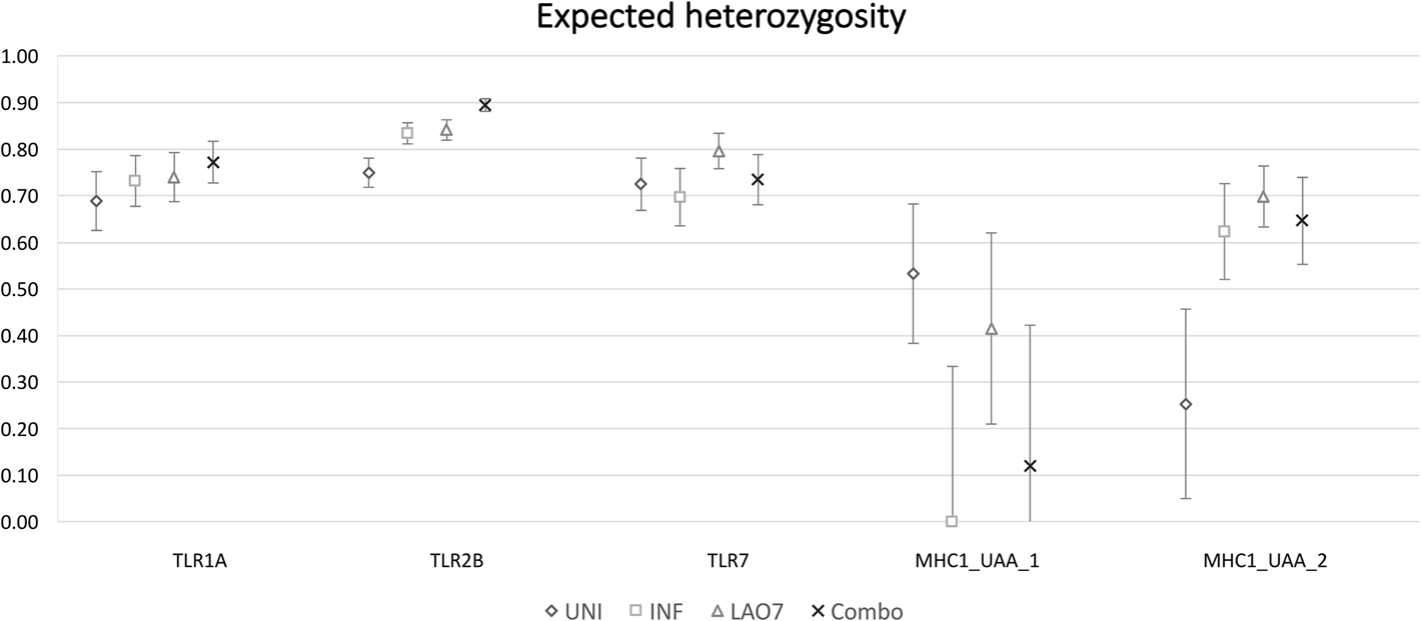
Expected heterozygosity for each locus and group. Error bars are +/− one standard error from the mean. *UNI* Uninfected group, *LA07* infected with LA07 strains, *INF* infected with various strains other than LA07. COMBO represents combined infected groups (the weighted average of LA07 and INF)

For each locus, contingency tables were constructed with the frequency of each allele in each of the three groups (UNI, LA07, INF) in order to test for associations between allele frequencies and groups (Chi-square, Fisher’s Exact Test, and Wilk’s G) and the statistical results are given in Additional file 1 (Table S4). In each case the null hypothesis that the allele frequencies and groups are independent was rejected at a significance level of α = 0.05. When comparing UNI vs. COMBO (the weighted average of LA07 and INF), the same premise held true: the allele frequencies are significantly different among groups. Charts comparing allele frequencies can be seen in Fig. 1 for the TLR loci and Fig. 2 for the MHC loci, and individual alleles within each locus and group whose frequencies are significantly higher or lower (i.e. above or below the 95% CI) than the expected frequencies based on the resampling method are indicated. Figure 3 indicates expected heterozygosity for each locus: standard error bars indicate there are differences in mean expected heterozygosity between groups at the TLR2B and MHC1-UAA-1 loci, with uninfected groups showing lower heterozygosity than infected groups.

#### Distance-based analyses

The results of the hierarchical AMOVA to determine sources of allele sequence variation are shown in Additional file 1 (Table S5). For all loci, the highest source of variation (77–90%) was within populations (i.e. the groups UNI, LA07, and INF), whereas the source of variation among the populations ranges from 0 to 22%. The source of variation among regions (i.e. the groups UNI and COMBO) ranges from 0 to 13% across loci. Phi-PT values (the sequence-based FST analogue) representing the pairwise differentiation at each locus for each pair of populations range from 0 to 0.299, and are in most cases significant.

## Discussion

We found that the allele frequencies at the three TLR and two MHC loci sequenced in this study, as well as the expected heterozygosities, are associated with infection status in the bananaquit. This result is to be expected if particular alleles and/or the overall number of alleles at these loci confer or contribute to pathogen resistance or susceptibility.

Parasite-mediated selection is thought to drive immune genes to evolve primarily under.

balancing selection, in which polymorphisms at these loci are maintained via negative frequency-dependent selection or heterozygote advantage (over-dominance) [25, 26]. Evidence for balancing selection has been shown in studies of MHC diversity in birds and other taxa [27–29]. These findings include birds exposed to avian malaria, which suggest that balancing selection is capable of maintaining MHC variation even in populations undergoing a genetic bottleneck [30]. Evidence for mechanisms underlying balancing selection are mixed. For example, in the mouse lemur, particular rare MHC alleles were found to be associated with resistance to nematode infection [31], whereas in the alpine ibex, MHC heterozygosity was associated with resistance to bacterial conjunctivitis [32]. Additionally, support for the “optimality hypothesis” has been shown: in the stickleback an intermediate level of MHC alleles was associated with resistance to tapeworms and parasitic fungi [33]. In the Chinese egret, both a particular MHC allele and an intermediate number of alleles was associated with infection status and burden by parasitic nematodes [34].

At TLR loci, evolution by balancing selection has been implicated in humans [35], and in bank voles both particular alleles of some TLRs and heterozygosity of other TLRs are associated with resistance to the blood pathogen *Bartonella* [36]. Evidence in other mammalian taxa, however, suggests directional selection is the dominant force [37]. Avian studies have indicated TLR genes evolve primarily under stabilizing or purifying selection attributed to functional constraints, with small portions of the gene under episodic positive selection for amino acid diversification [14, 16]. Studies of TLRs in avian species on a micro-evolutionary scale are rare, but research aimed at detecting balancing selection in populations of conservation concern indicate that genetic drift is the dominant force shaping their genetic variability [38, 39].

We found the presence and frequency of immune alleles at each locus show an overall significant trend in which infected individuals harbor some unique alleles and also have more alleles than uninfected individuals. The pattern is exemplified with TLR2B, in which the UNI group has 5 alleles ranging in frequency from 0.096–0.373, the LA07 group has 7 alleles (five shared with UNI and two additional ones) ranging from 0.008–0.234, and the INF group (OZ strains) has 7 alleles (all unique) ranging from 0.106–0.236.

This supports the idea that particular alleles confer resistance rather than high allelic diversity, and the lower frequency or absence of those alleles confer susceptibility. Among the five loci, the exception to this pattern is one region of the MHC1-UAA gene, MHC1-UAA-1, in which LA07 is fixed for one allele whereas the other groups have three alleles.

An advantage of the pooled sequencing approach is its relatively low cost and high efficiency [40], but it precludes the identification of genotypes within or among loci. Therefore observed zygosity cannot be determined, but the expected heterozygosity at each locus and group may give additional insight into mechanisms that may be operating, such as overdominance. In particular, with TLR2B and MHC1-UAA-2 locus, the mean expected heterozygosity is significantly lower in uninfected groups than in infected groups, which, taken along with the allele frequency data, suggests that heterozygote advantage is not operating to confer resistance at these loci.

The hierarchical AMOVA takes nucleotide sequence distances into consideration to estimate the biggest contributions to the genetic variance present in each locus: within the group itself, among the groups, or between the regions into which the groups fall (i.e. uninfected or infected). Our data supported the hypothesis that host TLR and MHC sequences diverge in the presence of parasites, although they showed different sources for this genetic variance.

Among the TLRs, no source of variation is seen among regions, but there is variation among groups, indicating that sequence variation between uninfected and infected individuals is attributable to the particular infection status (specialist LA07 vs. generalist OZ strains) and not infection as a whole. The highest contribution among groups is at locus TLR2B, where it accounts for 22% of the molecular variance. This can also be seen in the high pairwise differentiation values of UNI vs. INF (Phi-PT = 0.244) and LA07 vs. INF (Phi-PT = 0.184) at that locus. Additionally, the high molecular divergence between INF and the other groups reflects the previous findings of only unique alleles in that group at that locus. The other two TLR loci, TLR1A and TLR7, show among group variation contributions of 6–10% and significant pairwise Phi-PT values ranging from 0.036–0.091. Taken together these data support the idea that TLR sequence evolution occurs in response to selection pressure by pathogens, and different alleles respond to particular strains of *Haemoproteus*. This might be expected for adaptive immune system genes like MHC which evolve in response to novel pathogens, but innate TLRs are thought to be more general in the PAMPs they recognize (e.g. they are expected to recognize PAMPs conserved among protozoans, but not to differentiate between ligands of lineages within one genus).

Among the MHC-UAA loci, the region (uninfected or infected) accounted for 9–13% of the variation, and the group accounts for 0–14% of the variation. Pairwise Phi-PT values showed differentiation among populations ranging from significant values of 0.126–0.364. These data supported the hypothesis that host MHC sequences divergence is due to general infection status as well as to the infection status by lineage.

### Caveats

Some caveats exist regarding interpretation of our data. The underlying assumption to our analyses is the equimolar pooling of individual products for equal representation during sequencing. While our best efforts were made to ensure this is the case, technical error could be introduced during the experiment, such as from variation in fluorometric quantification of DNA or undetected sequencing errors. Therefore, while we expect our results to have biological significance, we cannot rule out potential impacts from technical error on the patterns we observed. Additionally, opportunistic sampling may not precisely reflect the alleles present in the entire population. For example, the striking pattern at the TLR2B locus of no overlap in alleles between the INF group (the smallest group) and the other groups could be impacted in part by technical error or effects of sampling. Additional studies with more sampling and sequencing (at these loci and others) would help to address these potential impacts and further explore the underlying biological mechanisms in the observed patterns.

Caution is to be used regarding the interpretation of the two loci (MHC1-UAA-1 and MHC1-UAA-2) composing regions of the MHC1-UAA gene. Although the vast majority of polymorphisms seen at the two loci in the MHC-UAA gene are contained in noncoding regions, these regions are expected to be in linkage disequilibrium with the rest of the gene. We can say that in general, the MHC data showed the same trend as the TLR data, which supports the hypothesis that particular alleles confer resistance and that heterozygote advantage is not operating. While our results suggest that particular alleles of TLRs confer resistance, rather than heterozygote advantage, they also do not contradict the “optimal” number of alleles theory. The diversity seen in the infected groups may be too high, and the smaller number of alleles in the uninfected groups may be the optimal number for parasite recognition without autoimmune consequences. However, we cannot determine if birds that were infected did not survive and thus were unrepresented in our study.

Additionally, since all bananaquits in this study were collected from the same time and place, we have assumed they were all equally likely to be exposed to hemosporidian parasites at the same rate. Thus our above interpretation of the data is based on the assumption that uninfected group of bananaquits are uninfected because their immunogenetic repertoire allowed them to recognize and clear *Haemoproteus* infections. Similarly we assume that the LA07 group does not have the immune alleles necessary to recognize or clear the LA07 strain of *Haemoproteus*, and the INF group’s immune genes do not allow for recognizing or clearing the OZ strains of *Haemoproteus*. An alternative way of interpreting the data would result if one were to start with the assumption that the uninfected groups are instead those individuals that have not been exposed to parasites (so the results of that group become uninformative for our purposes). In this scenario the infected groups are those that are infected but survive, so presumably have an immunogenetic repertoire that allows the individuals to live with chronic malaria (as oppose to dying during the acute stage). In this case, any increased allelic diversity in these immune genes (relative to birds that died) would result from balancing selection due to heterozygote advantage.

## Conclusions

Among TLRs we observed a large amount of alleles maintained overall in the population, but within individuals the presence of certain alleles at each locus seem to confer resistance, as indicated by being present in higher frequencies in the uninfected groups than the infected groups and the sequence divergence between the alleles of the groups. As postulated by the red queen hypothesis, pathogens evolve in response to the most common alleles in the host population [41, 42]. Therefore rare alleles confer a selective advantage to the host, and over time directional selection causes the previously common alleles become less common and the previously rare alleles become more common.

In this study these “rare alleles” are present at a high frequency in the uninfected groups but rarer in the infected groups. While a high amount of polymorphism is required at these loci in the population as a whole (as evident in the many additional alleles in the other groups), within individuals it is particular alleles which matter (those which are still able to recognize and respond to pathogens). This supports the notion of directional or frequency-dependent selection (as opposed to heterozygote advantage) in the individual. To our knowledge, this is the first study to demonstrate associations between TLR alleles and infection statuses in a wild avian population, and our data suggest that certain alleles confer resistance and may differentially recognize haemosporidian pathogens even at the sub-genus level.

## Methods

**Fig. 4:**
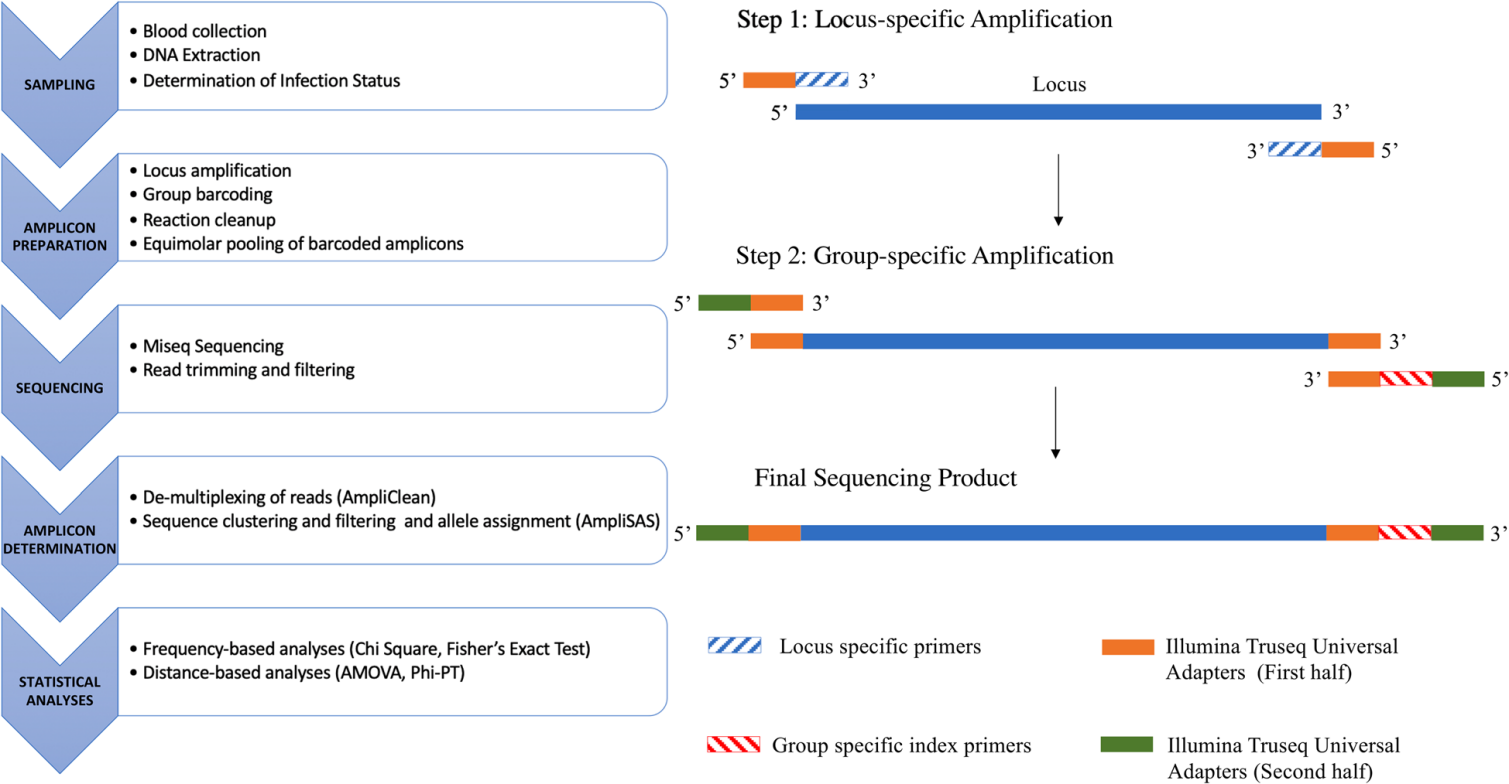
(Left) Overview of experimental design for investigating immune alleles among birds of different infection statuses using pooled sequencing of target amplicons (Right) Schematic of Primer Design

The experimental design for the investigation of immune gene alleles among bananaquits of different infection statuses using pooled sequencing of target amplicons is described below and summarized in Fig. 4.

### Sample collection

Bananaquit individuals were collected opportunistically along with other local avifauna via mist nets in Guanica Forest of Puerto Rico in 2001 as reported in [24]. Briefly, blood (5-10ul) from the brachial vein was drawn in the field and placed in Puregene cell lysis buffer, and genomic DNA was subsequently extracted by salt precipitation as reported in [24]. Animals were released upon blood collection. All applicable international, national, and institutional guidelines for the care and use of animals were followed: blood samples were collected and transported under the appropriate permits and licenses from local governments following protocols approved by the University of Pennsylvania and the University of Missouri, St Louis [24].

### Identification of infection status

In each individual, presence or absence of haemosporidian parasite infection was determined by PCR amplification of a conserved region of ribosomal rRNA as reported in [43]. For those that were infected, the lineage of *Plasmodium* spp. or *Haemoproteus* spp. was subsequently determined by amplification and sequencing of the mitochondrial cytochrome *b* using a variety of primer combinations as reported in [44].

### Pooled sequencing

The concentrations of the extracted genomic DNA from these previously sampled individuals with determined infection statuses were measured with a Nanodrop,spectrophotometer, and working solutions of 20 ng/ul were prepared. In order to create a library of pooled amplicons from all individuals at all loci we performed two PCR steps: the first to amplify the loci in all samples and the second to add Illumina flow-cell adapters and group-specific indexes to all samples. First we designed locus-specific primer sequences for portions of 12 immune (MHC and TLR) genes based on the annotated bananaquit genome [45]. For some genes (e.g. the MHC Class I gene MHC1-UAA), two sets of primers flanking different regions within the gene were chosen to increase our changes of capturing the peptide-binding region. The primers were chosen such that they flanked genomic regions containing putative ligand-binding sites and spanned no longer than 390 bp of the locus (to allow room for appending to the sequences for a 500-cycle MiSeq run).

We performed a two-step PCR to append Illumina adapters and unique indices to amplicons (Fig. 4, right panel). Specifically, we tailed the 5′ end of the locus-specific primers with 29–34 bp of the Illumina TruSeq Universal adapter sequences (Additional file 1: Table S2). Thus the first round of PCR amplified the loci (with the individuals in each experimental group in a separate plate) and created sequences which could be recognized by the primers in the next step of PCR. At this locus amplification step, if the locus did not amplify in the 99 individuals upon PCR optimization, that locus was disregarded from the study. The second PCR step incorporated the remaining Illumina Truseq Universal Adapter sequence and group index and includes the flow cell adapter sequence (Additional file 1: Table S2). In this way the locus-specific amplicons were labeled by experimental group (UNI = uninfected, LA07 = infected with host-specialist LA07 strain, or INF = infected with OZ02 and OZ21). See Additional file 1: Table S3 for PCR conditions.

The resulting products were cleaned using Sephadex columns and visualized by agarose gel electrophoresis. Each successful product (representing an amplicon from one locus from one individual) was quantified using an Invitrogen Quant-iT PicoGreen dsDNA assay kit, and read on a Neo Synergy plate fluorometer, in which the standard curve was replicated in triplicate and each plate was read three times and the values averaged.. The products were subsequently pooled equimolarly to a total of 10 nM. This library of pooled products was sequenced for paired-end reads using a 500-cycle Illumina Miseq.

### Sequence processing

*The raw reads (2x250bp) were merged with* BBmap (Bushnell, https://sourceforge.net/projects/bbmap), with an overlap of 12 bases and a minimum length of 30 bases. Cutadapt was used for adapter removal, and Trimmomatic was used to clip lower quality bases (less than Phred-20) from both the 5′ and 3′ [46, 47]. Using a custom script, we sorted the trimmed, merged, and quality-controlled reads into separate fastq files using the experimental group indexes.

### Amplicon and allele identification

We used AmpliSAS for amplicon identification and allele identification from the pooled sequencing reads [48]. Reads from each of the three experimental group’s fastq file were first processed using the associated program AmpliCLEAN to remove reads not corresponding to any amplicon (locus) based on locus-specific forward and reverse primer sequences. AmpliSAS was then used for each experimental group to a) de-multiplex reads into amplicons based on locus-specific primers; b) cluster amplicon sequences into potential alleles and artifacts (due to PCR or sequencing errors); and c) filter artifacts and assign true alleles. The amplicon read depths were set at a minimum of 1000 reads and the maximum allowed by AmpliSAS of 5000 reads. The maximum number of alleles to be considered per amplicon was 15. Clustering parameters for alleles and artifacts allowed for 1% substitution errors and 0.01% indel errors. Filtering parameters included a minimum allele frequency of 4%, and sequences that were chimeras from other major sequences were discarded.

We calculated summary statistics for each locus to quantify their overall genetic variation. These included the number of segregating sites, Watterson’s estimator Θ, and nucleotide diversity (π).

### Statistical analyses

For all subsequent analyses we compared allele frequency and molecular distance differences within and among the three groups (UNI, LA07, INF). In addition, we created a group named COMBO which combines the two groups with infected individuals into one group (LA07 + INF), using a weighted average to take into account sample size, in order to compare all uninfected with all infected individuals.

#### Frequency-based analyses

Allele frequencies for each locus and group were calculated from the AmpliSAS output by dividing the number of reads for a particular allele by the total number of reads for that amplicon, such that the frequency of all alleles for a particular locus per group summed to one. Thus the number of reads per amplicon (locus) per treatment group were used as allele frequencies. To test for allelic frequency differences among groups we used XLSTAT to compute several association tests against the null hypothesis of independence between the rows (the frequency of particular alleles) and columns (the experimental group) of the contingency tables at a significance level of α = 0.05. A Chi-square test for independence was performed and the strength of any effect was calculated by the association parameter Cramer’s V. We also performed a Fisher’s Exact Test on contingency tables small enough (< 5 × 3) for an exact *p*-value, and a Wilks’ G^2^ Likelihood-Ratio Test for independence.

To robustly determine particular alleles with significantly high or low frequencies, we implemented a randomized resampling technique in which the observed values of each allele for each group was compared to a null distribution of allele frequencies for each allele. The null distribution was created by assuming the weighted average of the three groups represent the allele frequency of the population, based on group sample sizes and parasite prevalence of the overall population. Group sample sizes were calculated as twice the number of diploid individuals in the group (the number of alleles represented in the pool): 2*n* = 90 for UNI, 2*n* = 82 for LA07, and 2n = 26 for INF. The parasite prevalence in the overall population during the sampling period averaged 34% for the LA07 strain and 15.75% for OZ strains (the INF group), with the remaining 50.25% of individuals uninfected [24]. We then generated 1000 simulated populations of 100 individuals in which each individual has two alleles at a locus. For each simulated population the expected allele frequency was calculated for each allele, and then a 95% confidence interval (CI) for allele frequency was estimated across all replicates. The CIs were interpreted as the null distribution, to which the observed allele frequencies were compared. If the observed value of an allele was within the CI, it was not significantly different from the null distribution; it was significantly high or low if the observed value was larger or smaller than the CI. Additionally, expected heterozygosity for each locus and group were calculated based on their allele frequencies, with +/− one standard error from the mean.

#### Distance-based analyses

For each locus we wished to identify molecular variance among the alleles present within and among experimental groups while taking into account the sample sizes of the groups. We again estimated the theoretical number of alleles present for a particular locus and group by multiplying the allele frequency by the sample size for the group (2* the number of diploid individuals in the group). The equimolar pooling of all individuals at all loci prior to sequencing was implemented in an effort to sequence all DNA molecules evenly and distribute noise produced during sequencing evenly among all individuals/groups/loci/alleles. Thus we make the assumption that any over-counting of these theoretical numbers is done so proportionately, and these theoretical numbers of alleles were used as a proxy for individual haplotypes in the population for Analysis of Molecular Variance (AMOVA).

We implemented AMOVA in GenAlEx with a hierarchical structure to represent the presence or absence of infection as well as infection by certain lineages [49, 50]. There were two broad “regions” (UNI and COMBO) and three “populations” (UNI and LA07 and INF). LA07 and INF were populations in the COMBO region, while UNI was the only population in the UNI region. Then we created a haploid distance matrix for calculation of Phi-Statistics (an F-Statistics analog for sequence data), on which AMOVA is based. We also calculated Phi-PT values for pairwise group comparisons based on 999 permutations as a measurement of genetic divergence among groups.

## Additional file

Additional file 1: **Supplementary Tables. Table S1.** Bananquit individual sample IDs and group name by infection status. Collected in Guanica Forest, Puerto Rico in 2001. **Table S2.** Locus- and group- specific primers (5′ - > 3′) for loci successfully amplified and sequenced to quality-control specifications. **Table S3.** PCR conditions for library preparation of the loci successfully used in this study, listed by volume (ul) out of a total reaction volume of 25ul. Working solution concentrations listed. Locus amplification thermocycler profile: initial denaturing for 94 (2 m), 35 cycles of 94 (30s), annealing temp (30s), and 72 (30s), and final extension of 72 (5 m). Group adapter/index thermocycler profile: initial denaturing for 94 (2 m), 30 cycles of 94 (30s), annealing temp (30s), and 72 (30s), and final extension of 72 (2 m). **Table S4.** Results of association/correlation tests at a significance of α = .05 on contingency tables of allele frequencies. **Table S5.** Distance-based analyses for molecular variance. AMOVA with hierarchical structure (Regions = UNI vs. COMBO (LA07 + INF); Populations = UNI vs. LA07 vs. INF). Input in GenAlEx is a haploid distance matrix (each nucleotide position represented as a site and coded for calculation of Phi-Statistics. For pairwise group comparisons, Phi-PT values are shown below the diagonal. Probability, P(rand > = data) based on 999 permutations is shown above diagonal. (DOCX 41 kb)

## List of abbreviations

AMOVA: Analysis of molecular variance
LRR: Leucine-rich repeat
MHC: Major histocompatibility complex
PAMPs: Pathogen-associated molecular patterns
TLR: Toll-like receptor
