# Supplementary material for: Immunogenetic response of the bananaquit in the face of malarial parasites
Source: BMC Evol Biol. 2019 May 22;19:107. doi: 10.1186/s12862-019-1435-y (PMC6529992; doi:10.1186/s12862-019-1435-y)
Supplement: Supplementary file 1 — Supplementary Tables. Table S1. Bananquit individual sample IDs and group name by infection status. Collected in Guanica Forest, Puerto Rico in 2001. Table S2. Locus- and group- specific primers (5′ - > 3′) for loci successfully amplified and sequenced to quality-control specifications. Table S3. PCR conditions for library preparation of the loci successfully used in this study, listed by volume (ul) out of a total reaction volume of 25ul. Working solution concentrations listed. Locus amplification thermocycler profile: initial denaturing for 94 (2 m), 35 cycles of 94 (30s), annealing temp (30s), and 72 (30s), and final extension of 72 (5 m). Group adapter/index thermocycler profile: initial denaturing for 94 (2 m), 30 cycles of 94 (30s), annealing temp (30s), and 72 (30s), and final extension of 72 (2 m). Table S4. Results of association/correlation tests at a significance of α = .05 on contingency tables of allele frequencies. Table S5. Distance-based analyses for molecular variance. AMOVA with hierarchical structure (Regions = UNI vs. COMBO (LA07 + INF); Populations = UNI vs. LA07 vs. INF). Input in GenAlEx is a haploid distance matrix (each nucleotide position represented as a site and coded for calculation of Phi-Statistics. For pairwise group comparisons, Phi-PT values are shown below the diagonal. Probability, P(rand > = data) based on 999 permutations is shown above diagonal. (DOCX 41 kb) [file 12862_2019_1435_MOESM1_ESM.docx]

**Additional file 1**

**SUPPLEMENTARY TABLES**

**Table S1.** Bananquit individual sample IDs and group name by infection status. Collected in Guanico Forest, Puerto Rico in 2001.

**Table S2.** Locus- and group- specific primers (5' -> 3') for loci successfully amplified and sequenced to quality-control specifications.

**Table S3.** PCR conditions for library preparation of the loci successfully used in this study, listed by volume (ul) out of a total reaction volume of 25ul. Working solution concentrations listed. Locus amplification thermocycler profile: initial denaturing for 94 (2m), 35 cycles of 94 (30s), annealing temp (30s), and 72 (30s), and final extension of 72 (5m). Group adapter/index thermocycler profile: initial denaturing for 94 (2m), 30 cycles of 94 (30s), annealing temp (30s), and 72 (30s), and final extension of 72 (2m).

**Table S4.** Results of association/correlation tests at a significance of α=.05 on contingency tables of allele frequencies.

Table S5. Distance-based analyses for molecular variance. AMOVA with hierarchical structure (Regions = UNI vs. COMBO (LA07 + INF); Populations = UNI vs. LA07 vs. INF). Input in GenAlEx is a haploid distance matrix (each nucleotide position represented as a site and coded for calculation of Phi-Statistics. For pairwise group comparisons, Phi-PT values are shown below the diagonal. Probability, P(rand >= data) based on 999 permutations is shown above diagonal. Significant values are bolded.

**Table S1**. **Bananaquit individual sample IDs and group name by infection status. Collected in Guanica Forest, Puerto Rico in 2001.**

| GF-41 | UNI | GSA-BANA-197 | UNI | GF-435 | LA07 |
| --- | --- | --- | --- | --- | --- |
| GF-50 | UNI | GSA-BANA-159 | UNI | GF-449 | LA07 |
| GF-237 | UNI | GSA-BANA-78 | UNI | GF-453 (#2) | LA07 |
| GF-227 | UNI | GSA-BANA-15 | UNI | GF-487 | LA07 |
| GF-189 | UNI | GSA-BANA-59 | UNI | GF-597 | LA07 |
| GF-185 | UNI | GSA-BANA-65 | UNI | GF-601 | LA07 |
| GF-183 | UNI | GSA-BANA-165 | UNI | GSA-BANA-20 | LA07 |
| GF-177 | UNI | GSA-BANA-155 | UNI | GSA-BANA-21 | LA07 |
| GF-176 | UNI | GSA-BANA-166 | UNI | GSA-BANA-95 | LA07 |
| GF-281 | UNI | GSA-BQ-107 | UNI | GSA-BANA-174 | LA07 |
| GF2-273 | UNI | GF-13 | LA07 | GSA-BANA-156 | LA07 |
| GF2-274 | UNI | GF-28 | LA07 | GSA-BANA-239 | LA07 |
| GF2-272 | UNI | GF-30 | LA07 | GSA-BANA-240 | LA07 |
| GF2-264 | UNI | GF-31 | LA07 | GSA-BANA-266 | LA07 |
| GF-255 | UNI | GF-34 | LA07 | GSA-BANA-268 | LA07 |
| GF-254 | UNI | GF-43 | LA07 | GSA-BANA-278 | LA07 |
| GF-251 | UNI | GF-44 | LA07 | GF-85 | INF (OZ21) |
| GF-249 | UNI | GF-45 | LA07 | GF-61 | INF (OZ21) |
| GF-248 | UNI | GF-46 | LA07 | GF-49 | INF (OZ21) |
| GF-243 | UNI | GF-54 | LA07 | GF-426 | INF (OZ21) |
| GF-241 | UNI | GF-64 | LA07 | GF-29 | INF (OZ21) |
| GF-238 | UNI | GF-66 | LA07 | GF2-275 | INF (OZ21) |
| GF-236 | UNI | GF-70 | LA07 | GF2-259 | INF (OZ21) |
| GF2-223 | UNI | GF-71 | LA07 | GF-172 | INF (OZ02) |
| GF-220 | UNI | GF-78 | LA07 | GF-167 | INF (OZ02) |
| GF-219 | UNI | GF-102 | LA07 | GSA-BANA-30 | INF (OZ02) |
| BSA-BANA-167 | UNI | GF-107 | LA07 | GF-436 | INF (OZ02) |
| GSA-BANA-139 | UNI | GF-161 | LA07 | GF-108 | INF (OZ02) |

Table S1 continued

| GSA-BANA-145 | UNI | GF-187 | LA07 | GF-105 | INF (OZ02) |
| --- | --- | --- | --- | --- | --- |
| GSA-BANA-138 | UNI | GF-217 | LA07 |  |  |
| GSA-BANA-133 | UNI | GF-218 | LA07 |  |  |
| GSA-BANA-130 | UNI | GF-223 | LA07 |  |  |
| GSA-BANA-125 | UNI | GF-246 | LA07 |  |  |
| GSA-BANA-121 | UNI | GF-428 (#2) | LA07 |  |  |
| GSA-BANA-08 | UNI | GF-431 | LA07 |  |  |

| **Table S2. Locus- and group- specific primers for loci successfully amplified and sequenced to quality-control specifications** | | | | |
| --- | --- | --- | --- | --- |
| **Primers for locus amplification (5' -> 3')** | | | |  |
| **Locus** | **TruSeq Universal Adapter** | **Locus-specific primers** |  | |
| TLR1A | F: AATGATACGGCGACCACCGAGATCTACAC | F: GAGCCGTATGAAATCCCTGA |  | |
|  | R:GTGACTGGAGTTCAGACGTGTGCTCTTCCGATCT | R: AGGAACACTTGAACGGGTTG |  | |
| TLR2B | F: AATGATACGGCGACCACCGAGATCTACAC | F: TGCTTGGCCATCACTACAAA |  | |
|  | R:GTGACTGGAGTTCAGACGTGTGCTCTTCCGATCT | R: GCTCCATTCTCCTGAAGGACT |  | |
| TLR7 | F: AATGATACGGCGACCACCGAGATCTACAC | F: TAGCCCATTTGAGGAAGCTG |  | |
|  | R:GTGACTGGAGTTCAGACGTGTGCTCTTCCGATCT | R: TTTCGGAGCATCAGTTCTTG |  | |
| MHC1-UAA-1 | F: AATGATACGGCGACCACCGAGATCTACAC | F: GTTCTCCACTCCCTGCGTTA |  | |
|  | R:GTGACTGGAGTTCAGACGTGTGCTCTTCCGATCT | R: ATCCCATCCCAGCCTAAAAG |  | |
| MHC1-UAA-2 | F: AATGATACGGCGACCACCGAGATCTACAC | F: TGGCTGTGATCTCCTGTCTG |  | |
|  | R:GTGACTGGAGTTCAGACGTGTGCTCTTCCGATCT | R: ATTCTATGGGAGTGGGATGG |  | |
| **Primers for adding adapters and indexing by group (5' -> 3')** | | | | |
|  | **TruSeq Universal Adapter** | **Group barcode** | **Portion of TruSeq Universal Adapter (back-half sequences)** | |
| F: All Groups | AATGATACGGCGACCACCGAGATCTACAC | None | TCTTTCCCTACACGACGCTC | |
| R: UNI | CAAGCAGAAGACGGCATACGAGAT | CGTGAT | GTGACTGGAGTTCAGACGTG | |
| R: LA07 | CAAGCAGAAGACGGCATACGAGAT | ATTGGC | GTGACTGGAGTTCAGACGTG | |
| R: INF | CAAGCAGAAGACGGCATACGAGAT | TACAAG | GTGACTGGAGTTCAGACGTG | |

**Table S3**. **PCR conditions for library preparation of the loci successfully used in this study, listed by volume (ul) out of a total reaction volume of 25ul. Working solution concentrations listed. Locus amplification thermocycler profile: initial denaturing for 94 (2m), 35 cycles of 94 (30s), annealing temp (30s), and 72 (30s), and final extension of 72 (5m). Group adapter/index thermocycler profile: initial denaturing for 94 (2m), 30 cycles of 94 (30s), annealing temp (30s), and 72 (30s), and final extension of 72 (2m).**

|  | | **Template** (~20ng/ul DNA for locus or PCR for group) | **Primers** (For both F and R) (10uM) | **ASS Buffer** (100mM Tris-Cl pH 8.9, 500 mM KCl, 0.5 mg/mL BSA) | **MgCl_2_** (25mM) | **dNTPs** (1.25mM) | **DMSO** | **Taq** (5,000 U/ml) | **H_2_0** | **Annealing Temp**  (deg C) |
| --- | --- | --- | --- | --- | --- | --- | --- | --- | --- | --- |
| TLR1A | Locus | 2 | 0.6 | 2.5 | 1.5 | 4 | 2.5 | 0.2 | 11.7 | 65.0 |
|  | UNI | 0.5 | 1.2 |  |  |  | 0 | 0.2 | 15.1 | 61.5 |
|  | LA07 | 0.5 | 0.4 |  |  |  | 2.5 | 0.2 | 13.4 | 61.5 |
|  | INF | 0.5 | 0.4 |  |  |  | 2.5 | 0.2 | 13.4 | 61.5 |
| TLR2B | Locus | 2 | 0.5 |  |  |  | 2.5 | 0.2 | 11.8 | 61.5 |
|  | UNI | 0.5 | 0.4 |  |  |  | 2.5 | 0.2 | 13.4 | 61.5 |
|  | LA07 | 0.5 | 0.4 |  |  |  | 2.5 | 0.2 | 13.4 | 61.5 |
|  | INF | 0.5 | 0.4 |  |  |  | 2.5 | 0.4 | 13.2 | 61.5 |
| TLR7-1 | Locus | 2 | 0.6 |  |  |  | 2.5 | 0.2 | 11.7 | 62.0 |
|  | UNI | 0.5 | 0.4 |  |  |  | 2.5 | 0.2 | 13.4 | 61.5 |
|  | LA07 | 0.5 | 0.4 |  |  |  | 2.5 | 0.2 | 13.4 | 61.5 |
|  | INF | 0.5 | 0.4 |  |  |  | 2.5 | 0.2 | 13.4 | 61.5 |
| MHC1-UAA-1 | Locus | 3 | 1.2 |  |  |  | 2.5 | 0.2 | 10.1 | 64.5 |
|  | UNI | 0.5 | 0.4 |  |  |  | 2.5 | 0.2 | 13.4 | 61.5 |
|  | LA07 | 0.5 | 0.5 |  |  |  | 2.5 | 0.2 | 13.3 | 61.5 |
|  | INF | 0.5 | 0.4 |  |  |  | 2.5 | 0.2 | 13.4 | 61.5 |
| MHC1-UAA-2 | Locus | 2 | 0.6 |  |  |  | 2.5 | 0.2 | 11.7 | 61.5 |
|  | UNI | 0.5 | 0.4 |  |  |  | 2.5 | 0.2 | 13.4 | 61.5 |
|  | LA07 | 0.5 | 0.5 |  |  |  | 2.5 | 0.2 | 13.3 | 61.5 |
|  | INF | 0.5 | 0.4 |  |  |  | 2.5 | 0.2 | 13.4 | 61.5 |

**Table S4**. **Results of association/correlation tests at a significance of α=.05 on contingency tables of allele frequencies.**

|  | **UNI VS. LA07 VS. INF** | | | | |
| --- | --- | --- | --- | --- | --- |
|  | TLR1A | TLR2B | TLR7 | MHC-UAA-1 | MHC-UAA-2 |
| Chi-square (Obs value) | 134.289 | 341.331 | 63.981 | 45.346 | 54.364 |
| Chi-square (Crit value) | 21.026 | 38.885 | 21.026 | 9.488 | 12.592 |
| DF | 12 | 26 | 12 | 4 | 6 |
| p-value | < 0.0001 | < 0.0001 | < 0.0001 | < 0.0001 | < 0.0001 |
| Cramer's V (association coeff) | 0.473 | 0.754 | 0.327 | 0.275 | 0.301 |
| Fisher's Exact Test p-value | N/A | N/A | N/A | < 0.0001 | < 0.0001 |
| Wilks' G² (Obs value) | 167.653 | 417.306 | 70.165 | 64.117 | 71.447 |
| Wilks' G² (Critical value) | 21.026 | 38.885 | 21.026 | 9.488 | 12.592 |
| DF | 12 | 26 | 12 | 4 | 6 |
| p-value | < 0.0001 | < 0.0001 | < 0.0001 | < 0.0001 | < 0.0001 |
|  |  |  |  |  |  |
|  | **UNI VS. COMBO** | | | | |
|  | TLR1A | TLR2B | TLR7 | MHC-UAA-1 | MHC-UAA-2 |
| Chi-square (Obs value) | 34.530 | 53.266 | 17.471 | 29.403 | 37.559 |
| Chi-square (Crit value) | 12.592 | 22.362 | 12.592 | 5.991 | 7.815 |
| DF | 6 | 13 | 6 | 2 | 3 |
| p-value | < 0.0001 | < 0.0001 | 0.008 | 0.383 | 0.316 |
| Cramer's V (association coeff) | 0.415 | 0.516 | 0.296 | < 0.0001 | < 0.0001 |
| Fisher's Exact Test p-value | < 0.0001 | < 0.0001 | 0.005 | < 0.0001 | < 0.0001 |
| Wilks' G² (Obs value) | 43.553 | 68.713 | 19.496 | 31.971 | 49.067 |
| Wilks' G² (Critical value) | 12.592 | 22.362 | 12.592 | 5.991 | 7.815 |
| DF | 6 | 13 | 6 | 2 | 3 |
| p-value | < 0.0001 | < 0.0001 | 0.003 | < 0.0001 | < 0.0001 |

Table S5. Distance-based analyses for molecular variance. AMOVA with hierarchical structure (Regions = UNI vs. COMBO (LA07 + INF); Populations = UNI vs. LA07 vs. INF). Input in GenAlEx is a haploid distance matrix (each nucleotide position represented as a site and coded for calculation of Phi-Statistics). For pairwise group comparisons, Phi-PT values are shown below the diagonal. Probability, P(rand >= data) based on 999 permutations is shown above diagonal.
